# Different leafy vegetable cropping systems regulate growth, photosynthesis, and PSII functioning in mono-cropped eggplant by altering chemical properties and upregulating the antioxidant system

**DOI:** 10.3389/fpls.2023.1132861

**Published:** 2023-04-18

**Authors:** Muhammad Imran Ghani, Ahmad Ali, Muhammad Jawaad Atif, Muhammad Ali, Mohammad Abass Ahanger, Xiaoyulong Chen, Zhihui Cheng

**Affiliations:** ^1^College of Agriculture, Guizhou University, Guiyang, China; ^2^Key Laboratory of Karst Georesources and Environment, Ministry of Education, College of Resources and Environmental Engineering, Guizhou University, Guiyang, China; ^3^College of Horticulture, Northwest A&F University, Yangling, China; ^4^Horticultural Research Institute, National Agricultural Research Centre, Islamabad, Pakistan; ^5^College of Life Sciences, Northwest A&F University, Yangling, China; ^6^College of Ecology and Environment, Tibet University, Lhasa, Tibet, China

**Keywords:** continuous cropping, crop rotation, sustainable vegetable production, lipid peroxidation, plant defense system, soil available nutrients, eggplant

## Abstract

Continuous cropping of eggplant threatened regional ecological sustainability by facilitating replanting problems under mono-cropping conditions. Therefore, alternative agronomic and management practices are required to improve crop productivity at low environmental cost for the development of sustainable agricultural systems in different regions. This study examined changes in soil chemical properties, eggplant photosynthesis, and antioxidant functioning in five different vegetable cropping systems over a 2-year period., 2017 and 2018. The results showed that welsh onion-eggplant (WOE), celery-eggplant (CE), non-heading Chinese cabbage-eggplant (NCCE), and leafy lettuce-eggplant (LLE) rotation systems significantly impacted growth, biomass accumulation, and yield than fallow-eggplant (FE). In addition, various leafy vegetable cropping systems, WOE, CE, NCCE, and LLT induced significant increases in soil organic matter (SOM), available nutrients (N, P, and K), and eggplant growth by affecting the photosynthesis and related gas exchange parameters with much evident effect due to CE and NCCE. Moreover, eggplant raised with different leafy vegetable rotation systems showed higher activity of antioxidant enzymes, resulting in lower accumulation of hydrogen peroxide and hence reduced oxidative damage to membranes. In addition, fresh and dry plant biomass was significantly increased due to crop rotation with leafy vegetables. Therefore, we concluded that leafy vegetable crop rotation is a beneficial management practice to improve the growth and yield of eggplant.

## 1 Introduction

The consistent increase in the global population has increased the demand for food and cash crops. Due to limited agricultural land worldwide and the decreasing number of new crop areas, monoculture is a common model for large-scale, intensive agricultural production, especially in the horticultural industry (Tan et al., 2021; Bhatti et al., 2022). Even with a good field management regime, the crop may still experience growth and yield reduction and promote disease incidence (Zeng et al., 2020). Mono-cropping refers to the practice of growing the same type of crop every year for a long period of time (Scarascia-Mugnozza et al., 2011; Xiao et al., 2012). The overuse of synthetic fertilizers and agrochemicals is inevitable in the mono-cropping system, which leads to mono-cropping obstacles (Ghani et al., 2019b; Ali et al., 2021; Ghani et al., 2022b). The mono-cropping obstacles are attributed to soil salinization, acidification, nutrient imbalance, and autotoxicity (Lyu et al., 2020; Zeng et al., 2020).

Eggplant (*Solanum melongena* L.) is a valuable vegetable cash crop mostly grown under plastic shed (Wang et al., 2015; Ghani et al., 2022a). Eggplant production in plastic shed heavily relies on mono-cropping systems. Like other crops, consecutive eggplant cultivation could occur mono-cropping obstacles, including an upsurge in autotoxins in the soil, which hampers plant growth and development, reduces resistance to harsh environmental conditions, and ultimately reduces plant yield and quality (Wang et al., 2015). One of the beneficial practices that ameliorate the negative impact of mono-cropping is crop rotation. To enhance productivity and optimize the profitability of a rotation system, rotated crops should be appropriately selected (Li et al., 2017; Ali et al., 2019). Diversified crop rotation during the fallow period mitigates the adverse effects of mono-cropping obstacles by sustaining the soil quality via nutrient deposit (St. Luce et al., 2020), greater SOM input (Ali et al., 2021), soil carbon sequestration (Song et al., 2018), and minimizing pest and disease attacks, particularly compared with mono-cropping systems (Ali et al., 2021; Ghani et al., 2022b). Different leafy vegetable plants have legacy effects on soil through root exudation, suppress soil-borne pathogens, and enhance soil fertility, improving plant growth and yield of the subsequent crop (Ali et al., 2019; Ghani et al., 2022b). Numerous studies have demonstrated yield advantages of crop rotation to subsequent crops, including tomato-onion, tomato-chrysanthemum, hairy vetch-eggplant, and cow pea-broccoli significantly improved tomato, eggplant, and broccoli yield compared with mono-cropping planting (Tian et al., 2009; Radicetti et al., 2016; Sánchez-Navarro et al., 2020). Furthermore, crop rotation also enhanced plant tolerances to different types of stresses (Gaudin et al., 2015).

Plants might be subjected to a wide range of external stresses during their growth period, including salt and heat stress and water deficit. However, crops grown in mono-cropping suffer from additional stresses such as evolving diseases and pests, various biotic and abiotic stresses, reduced soil physical-chemical characteristics, and the gradual buildup of root exudates in the soil. All of these stresses pose a constant threat to plant growth. (Chen et al., 2011; Wang et al., 2015). Moreover, physiological and biochemical alterations following mono-cropping involve reduced growth underlined by a significant decline in photosynthetic rate. In addition, changes in soil pH adversely affect growth by declining photosynthesis and gas exchange parameters (Long et al., 2017). During stressful conditions, plants promote the production of reactive oxygen species (ROS) and cause significant damage to DNA, lipid peroxidation, the cell membrane, and proteins However, plants develop efficient adaptive strategies such as generating antioxidants, secondary metabolite, and osmolyte metabolism to overcome these stresses (Ahanger and Agarwal, 2017b; Ahanger et al., 2017; Ghani et al., 2022c). These biochemical pathways serve as an early signaling molecule of the plant’s defense response to a variety of environmental stresses and as a secondary messenger for subsequent defense reactions (Yin et al., 2015; Chen et al., 2022). Increased activity of the antioxidant system and the metabolism of secondary metabolites avoid oxidative damage to membranes and other important macromolecules in the cell, hence keeping important mechanisms such as photosynthesis from being disrupted (Ahanger et al., 2017; Ahanger et al., 2018)

Previous studies have figure out the importance of crop rotation on pathogen suppression and soil stability (Thorup-Kristensen et al., 2012; Tian et al., 2013; Ali et al., 2019). More recently, we reported that leafy vegetables used as a crop rotation and their residue retention (above and below-ground biomass) significantly influenced soil nitrogen, microbial biomass, and soil enzymatic activity (Ghani et al., 2022b). However, limitation may still exist that dead roots and leaves were maintained in the field and well incorporated into the soil, and then influence on plant growth and development, as well as plant resistance-related enzymes. In this study, we postulated that executing different leafy vegetable plants would help to increase the eggplant’s production capacity. Therefore, the aim of the study was to evaluate the capacities of different leafy vegetables, welsh onion, celery, non-heading Chinese cabbage, and leafy lettuce to alleviate mono-cropping obstacles of eggplant cultivation that generally occur due to mono-cropping, as well as to determine a sustainable vegetable cropping system to enhance eggplant production. Hence, the influence of different leafy vegetables on soil chemical properties, plant morphological and physiological observations, lipid peroxidation (MDA), and H_2_O_2_ level and, correlations between plant growth, physiology, and soil chemical properties were assessed.

## 2 Material and methods

### 2.1 Experimental site description and experimental design

Two years of field experiment under a plastic shed was conducted at the research station of Northwest A&F University Yangling, China. From March 2013 to November 2016, the eggplant was continuously cultivated under this plastic shed for four years. The eggplant was cultivated once a year with a mono-cropping regime. The growing season of eggplant starts from 1^st^ week of March to mid-November. The plastic shed’s soil is sandy loam soil. The specific details of basic soil properties were reported previously by (Ghani et al., 2022b). After four years of consecutive eggplant cultivation, a rotational experiment was performed with four different winter leafy vegetables, including (I) welsh onion (Allium fistulosum L.), (II) celery (Apium graveolens L.), (III) non-heading Chinese cabbage (Brassica rapa L.), and (IV) leafy lettuce (Lactuca sativa L.) during the fallow period of eggplant from mid-November to 1^st^ week of March. These leafy vegetables were harvested at the full leaf growth stage in 1^st^ week of March, followed by the immediate planting of eggplant. The leafy vegetables root left over were mixed in the soil before eggplant seedlings were transplanted. With a factorial randomized complete block design, the eggplant seedlings were transplanted to the field in both years on the second week of March and harvested on 2^nd^ week of November in both years. The experiment consisted of five different planting systems with three replications: fallow–eggplant (FE), welsh onion–eggplant (WOE), celery–eggplant (CE), no-heading Chinese cabbage–eggplant (NCCE), and leafy lettuce–eggplant (LLE) with three replications.

Each cropping system and fellow eggplant was assigned three plots and each plot consist of three beds. The size of each plot was 12.60 m^2^ (3.6 m long . 3.5 m wide) and each bed was 4.20 m^2^ (3.5 m long . 1.2 m wide). A thin, impermeable plastic sheet was inserted at a depth of 50 cm into the soil among experimental plots and extended to 5 cm above the ground, intended to prevent the passage of water and nutrients between plots and stop the interplay of various treatments. Three-week-old uniform eggplant seedlings (*Solanummelongena* L. Cv.Tai Kong Qie Wang) with 3 leaves were transplanted to the above-prepared beds; each bed consists of two rows. There were 7 seedlings in each row, and 14 in each bed, with 0.8 m distance between rows and 0.5 m between plants. Each year, before eggplant planting, each bed was fertilized with organic fertilizer (PengDiXin) at the rate of (50.65 kg ha^−1^), “SaKeFu” (119.04 kg ha^−1^). The detailed information is previously reported in (Ghani et al., 2022b). In addition, JinBa fertilizer was top-dressed (0.5kg/bed) according to local recommendations for vegetable production. No chemical fertilizers were used during the winter leafy vegetable growth period, and the same amount of irrigation, fertilization, and management practices were carried out throughout the experiment.

### 2.2 Measurement of morphological indices

To evaluate morphological traits at different growth phases, we randomly sampled three plants from each replication and 9 plants from each treatment. The growth phases included the first flowering, the first fruiting, the second flowering, and the second fruiting. A measuring tape was utilized to get an accurate reading of the plant height. The diameter of the stem was determined using an electronic vernier caliper. The eggplant’s fresh weight (FW) was measured using an electronic scale after the eggplant was harvested, whereas the eggplant’s dry weight (DW) was determined after oven drying at 70 °C for 72 hours.

### 2.3 Quantification of photosynthetic pigment and gas exchange parameters

Chlorophyll a, b, total chlorophyll, and carotenoids were measured by placing 0.5 g of fresh leaf tissue into a 25 mL glass tube with 20 mL 80% acetone for 48 hours in the dark after that, and absorbance was determined spectrophotometrically (UV-3802, UNICO, MDN, USA) at 645 nm, 663 nm, and 652 nm, respectively (Arnon, 1949).

Net photosynthetic rate (pN), stomatal conductance (gs), intercellular CO_2_ concentration (Ci), and transpiration rate (E) were measured in the uppermost leaf by using a LI-6400 portable photosynthesis system (Li-Cor, Lincoln, NE, USA).

Measurement of the maximal photochemical efficiency (Fv/Fm), photosystem II (ФPSII), non-photochemical quenching coefficient (NPQ) and, photochemical quenching (qP) were determined using modulated chlorophyll fluorometer (PAM-2000 chlorophyll fluorometer) after 20 min of dark adaptation. The recorded data were processed by PAM Win software.

### 2.4 Antioxidant enzymes assay

The eggplant leaves (0.5 g) were homogenized in a chilled 0.05 mM (pH 7.8) phosphate buffer containing 0.1% polyvinylpyrrolidone and 0.5 M ethylenediaminetetraacetic acid (EDTA). The homogenate was centrifuged at 12000 g for 15 min at 4°C, and the supernatant was used for enzyme analysis. To estimate superoxide dismutase (SOD) activity, we followed the method of (Dhindsa et al., 1981). The enzyme’s ability to inhibit the photochemical reduction of nitroblue tetrazolium (NBT) was monitored at 560 nm.

The reaction mixture was prepared by adding 0.5 mL enzyme extract into 50 mL of 0.05 M phosphate buffer (pH 7.8), 28 mL guaiacol, and 19 mL 30% H_2_O_2_ (v/v) were mixed. The prepared reaction mixture of 3.5 mL was then transferred to a cuvette (1 cm) path length. An increase in absorbance was recorded at 470 nm wavelength over 3 min at 30-s intervals.

For the Catalase (CAT) activity, an enzyme extract of (0.1 mL) was added to 1.9 mL of reaction mixture containing phosphate buffer 200 mM (pH 7.0) and 1 mL of 0.3% H_2_O_2_. The enzyme activity was assessed by observing the decrease in absorbance at 240 nm for 2 min (Chance and Maehly, 1955).

Ascorbate peroxidase (APX) extraction was quantified by observing a reduction in absorbance due to the oxidation of ascorbic acid at 290 nm according to the method of (Nakano and Asada, 1981). The enzyme mixture consisted of 50 mM potassium phosphate buffer (pH 7.0), 0.2 mM EDTA, 0.5 mM ascorbic acid, 2 mM H_2_O_2_, and 100 μL enzyme extract.

Polyphenol oxidase (PPO) activity was assayed by measuring the initial increase in absorbance during the first 3 min of the reaction at 410 nm (Zheng et al., 2007). PAL activity was assessed according to the method of (Gao, 2006).

### 2.5 Measurement of oxidative stress biomarkers

The concentration of H_2_O_2_ was estimated following the method of (Velikova et al., 2000). For lipid peroxidation, the content of malonaldehyde (MDA) was measured by incubating tissue extract with thiobarbituric acid (TBA) at boiling temperature (Dhindsa et al., 1981).

### 2.6 Assessment of protein content

The protein content was quantified using the method proposed by (Bradford, 1976) using BSA as a standard.

### 2.7 Measurement of soil chemical properties

Soil organic matter (SOM) was determined through the procedure described by (Walkley and Black, 1934). We utilized the alkali-hydrolyzed diffusion method to determine the amount of available nitrogen (AN) (Shi, 1996). The development of a complex that was blue in colour following the extraction of 0.5 M NaHCO3 at a pH of 8.5 was used to measure the amount of accessible phosphorus (AP) in the soil (Murphy and Riley, 1962). The following procedure was used to analyze the available K (Knudsen et al., 1983).

### 2.8 Statistical analysis

Data presented are the mean of three replicates and were statistically analyzed by two-way analysis of variance (ANOVA) as a 5 × 2 (treatment × year) factorial design for the experiment, and Tukey HSD test was used to analyze the mean separations among treatments at p < 0.05. Using origin software, a Pearson correlation was conducted to evaluate the relationship between plant growth, physiological traits, and soil chemical properties. All statistical analyses were performed with SPSS v.19.0 (SPSS Inc., Chicago, USA).

## 3 Results

### 3.1 Effect of different leafy vegetable cropping systems on morphological traits and chlorophyll pigments in eggplant leaves

Results illustrated in **Table 1** showed that plant height and stem diameter were significantly (*P <* 0.05) affected by WOE, CE, NCCE and, LLE cropping systems as compared to FE. The highest plant height was observed for NCCE and LLE cropping systems than FE, and the observed increase was 18.34% and 16.15%, respectively. Regarding the growing stages, the eggplant height showed a rapid increase from 1^st^ flowering to 2^nd^ flowering stage and then slowed down at later stages. The maximum increase was recorded at 2^nd^ fruiting stage (297.38%) than other growth stages, whereas the year factor exhibited a non-significant effect. Maximum stem diameter was observed for CE as compared to FE, and the increase was 15.54%. Regarding growth stages, stem diameter increased gradually from 1^st^ flowering to 2^nd^ flowering. The maximum stem diameter was observed at 2^nd^ fruiting stage as compared to other growth stages, and the increase was 45.32%. The year factor also had a significant effect, and the highest increase in stem diameter was observed in 2018 as compared to 2017 (**Table 1**).

**Table 1:** Effect of different leafy vegetable rotation systems on morphological indexes and photosynthetic pigments of eggplant at different growth stages during the years 2017 and 2018.

Treatment	Plant height (cm)	Stem diameter (mm)	Chlorophyll a (mg g^-1^ FW)	Chlorophyll b (mg g^-1^ FW)	Chlorophyll ab (mg g^-1^ FW)	Carotenoids (mg g^-1^ FW)
Year						
2017	111.1 ± 48.57a	14.200 ± 2.64b	17.18 ± 2.44b	6.77 ± 2.23b	24.42 ± 2.90b	2.96± 1.35b
2018	112.2 ± 48.32a	14.771 ± 2.79a	18.16 ± 2.46a	7.63 ± 2.02a	25.63 ± 2.66a	3.69 ± 2.66a
Leafy vegetables						
FE	100.3 ± 43.95c	13.941 ± 2.33c	16.84 ± 2.36d	6.18 ± 1.95e	23.90 ± 3.05d	2.87± 1.23d
WOE	107.1 ± 41.35b	15.196 ± 2.41b	16.98 ± 2.34d	6.68 ± 1.96d	24.49 ± 2.82c	3.19 ± 1.46c
CE	115.8 ± 51.16a	16.107 ± 2.30a	18.00 ± 2.27b	7.73 ± 2.01b	25.74 ± 2.68a	3.57 ± 1.51ab
NCCE	118.7 ± 52.01a	14.906 ± 2.62b	19.11 ± 2.74a	8.36 ± 1.99a	26.10 ± 2.30a	3.62 ± 1.49a
LLE	116.5 ± 50.31a	12.275 ± 2.32d	17.41 ± 2.04c	7.05 ± 2.22c	24.89 ± 2.77b	3.40± 2.77bc
Stage						
1^st^ flowering	39.81 ± 3.69d	12.145 ± 1.30c	20.81 ± 1.29a	9.80 ± 1.03a	28.48 ± 0.88a	5.50 ± 0.62a
1^st^ fruiting	97.95 ± 6.37c	12.509 ± 1.34c	18.57 ± 0.88b	8.10 ± 0.95b	26.14 ± 0.85b	3.40 ± 0.82b
2^nd^ flowering	150.8 ± 11.34b	15.636 ± 1.68b	16.56 ± 1.02c	6.22 ± 2.01c	24.10 ± 1.19c	2.47 ± 0.28c
2^nd^ fruiting	158.2 ± 12.34a	17.650 ± 1.66a	14.73 ± 0.98d	4.68 ± 1.99d	21.37 ± 1.50d	1.94 ± 0.44d
F-test						
Year (Y)	ns	***	***	***	***	***
Leafy vegetable (LV)	***	***	***	***	***	***
Stage (S)	***	***	***	***	***	***
Y × LV × S	ns	ns	*	**	*	*

Data are presented as means with standard deviation (n= 9). Different letters show significant difference at p<0.05 level. FE, fellow eggplant; WOE, welsh onion-eggplant: CE, celery-eggplant; NCCE, non-heading Chinese cabbage-egglant; LLE, leafy lettuce-eggplant. *p < 0.05; **p < 0.01; ***p < 0.001; ns, non-significant.

Leafy vegetable cropping systems significantly stimulated the chlorophyll pigments. Maximum chlorophyll a content was observed for NCCE as compared to FE rotation, and the increase was 13.48% (**Table 1**). With respect to growth stages, maximum content of chlorophyll a was recorded at 1^st^ flowering stage as compared to other growth stages, and the increase was 41.27%, and the minimum chlorophyll a was observed at 2^nd^ fruiting stage. Year factor was also significant, and maximum chlorophyll a was observed in 2018. The interaction effect of three factors (Y × T × S) on chlorophyll a was also significant. Winter leafy vegetables significantly affected chlorophyll b pigment. The highest increase (35.27%) in chlorophyll b content was observed for NCCE as compared to FE rotation. Regarding growing stages, maximum chlorophyll b was observed at 1^st^ flowering stage, and an increase was 52.24%, and minimum chlorophyll b was observed at 2^nd^ fruiting stage. Year factor was also significant, and maximum chlorophyll b was observed in 2018. The interaction effect of three factors (Y × T × S) on chlorophyll b was also significant (**Table 1**).

It is shown in **Table 1** that chlorophyll a and b were significantly (*P <* 0.05) affected by leafy vegetable rotation systems. It was shown that the highest chlorophyll ab were recorded for NCCE, followed by CE as compared to FE, and an increase was 9.20% and 7.69%, respectively. Chlorophyll ab were observed maximum at 1^st^ flowering stage, and an increase was 33.27%, and the minimum increase (24.96%) was observed at the 2^nd^ fruiting stage. Year factor was also significant, and maximum chlorophyll ab was observed in 2018. The interaction effect of three factors (Y × T × S) on chlorophyll ab was also significant.

The effect of different leafy vegetable species on carotenoid content is illustrated in **Table 1**. Leafy vegetables significantly (*P <* 0.05) enhanced the carotenoid contents compared to FE, and a significant effect of NCCE was observed on carotenoid content. Maximum carotenoid content was observed for NCCE as compared to FE, and the increase was 26.7%. The growth stage also had a significant effect on carotenoid content. The highest increase in carotenoid content was observed at the 1st flowering stage, and the lowest increase was observed at the 2^nd^ fruiting stage. The year factor was significant, and the highest carotenoid content was observed in 2018.

### 3.2 Effect of different leafy vegetable cropping systems on gas exchange parameters

Leafy vegetable cropping systems significantly (*P <* 0.05) affected gas exchange parameters (**Table 2**). pN was higher for CE and NCCE than FE, with an increase of 15.28% and 11.18%, respectively. Similarly, growth stages also had a significant effect on net photosynthesis. pN increased at all stages, and the maximum increase was observed at 1^st^ flowering stage, which was 39.94%, while the minimum increase was observed at 2^nd^ fruiting stage, which was 28.54%. Year factor was also significant, and maximum pN was observed in 2018. The interaction effect of three factors (Y × T × S) on pN was also significant (**Table 2**). In addition, leafy vegetable species had a significant impact on Ci. Ci was increased in all vegetable species as compared to FE. NCCE and CE exhibited higher Ci than FE, and the increase was 11.15% and 10.17%, respectively. The Ci increased at all growth stages, with the highest values recorded at 1^st^ flowering stage. The year factor had a non-significant effect on Ci (**Table 2**).

**Table 2:** Effect of different leafy vegetable rotation systems on gas exchange parameters of eggplant at different growth stages during the year 2017 and 2018.

Treatment	(pN) (µmol m^-2^ s^-1^)	Ci (µmol mol^-1^)	E (mmol m^-2^ s^-1^)	gs (mol m^-2^ s^-1^)
Year				
2017	26.50 ± 3.40a	281.0 ± 30.04a	0.029 ± 0.171a	1.38 ± 0.21a
2018	25.37 ± 3.55b	280.2 ± 19.39a	0.008 ± 0.006b	1.34 ± 0.22a
Leafy vegetables				
FE	23.88 ± 2.90d	263.5 ± 26.37d	0.006 ± 0.006c	1.13 ± 0.15d
WOE	25.52 ± 3.09c	271.7 ± 25.06c	0.007 ± 0.006b	1.28 ± 0.13c
CE	27.53 ± 3.59a	290.3 ± 22.29ab	0.008 ± 0.007a	1.43 ± 0.18b
NCCE	26.55 ± 3.76b	292.9 ± 19.22a	0.009 ± 0.007a	1.61 ± 0.13a
LLE	26.18 ± 3.11b	284.5 ± 19.42b	0.007 ± 0.006b	1.36 ± 0.13c
Stage				
1^st^ flowering	30.13 ± 1.98a	308.0 ± 8.98a	0.019 ± 0.002a	1.40 ± 0.21a
1^st^ fruiting	27.32 ± 1.58b	287.5 ± 15.60b	0.003 ± 0.001c	1.40 ± 0.15a
2^nd^ flowering	24.75 ± 1.24c	269.1 ± 15.45c	0.004 ± 0.001b	1.38 ± 0.22a
2^nd^ fruiting	21.53 ± 1.23d	257.8 ± 23.32d	0.004 ± 0.001b	1.26 ± 0.24b
F-test				
Year (Y)	***	ns	***	ns
Leafy vegetable (LV)	***	***	***	***
Stage (S)	***	***	***	***
Y × LV × S	ns	ns	**	ns

Main effect due to treatment (crop rotation), sampling year and their interaction was analyzed by Two-way ANOVA. Data are presented as means with standard deviation (n= 9). Different letters show significant difference at p<0.05 level. pN, net photosynthesis; Ci, internal CO_2_ rate; E, transpiration rate. gs, stomatal conductance; FE, fellow eggplant; WOE, welsh onion-eggplant: CE, celery-eggplant; NCCE, non-heading Chinese cabbage-egglant. **p < 0.01; ***p < 0.001; ns, non-significant.

Leafy vegetable rotations also had a significant (*P <* 0.05) effect on gs and E. The highest increase in gs and E was observed in NCCE as compared to FE, and the increase was 50% and 42.47%, respectively (**Table 3**). Similarly, growth stages also significantly affected gs and E. Maximum gs was observed at the 1st flowering stage with an increase of 375%, and maximum E was observed at the 1^st^ flowering stage 11.11%. The year factor had a non-significant effect on gs. Whereas, for E year factor was significant and maximum E was observed in 2018 (**Table 2**).

**Table 3:** Effect of different leafy vegetable rotation systems on chlorophyll fluorescence parameters of eggplant at different growth stages during the year 2017 and 2018.

Treatment	Fv/Fm	ФPSII	NPQ	qP
Year				
2017	0.73 ± 0.03a	0.70 ± 0.03a	0.34 ± 0.02a	0.79 ± 0.03b
2018	0.71 ± 0.03b	0.66 ± 0.03b	0.26 ± 0.02b	0.83 ± 0.06a
Leafy vegetables				
FE	0.70 ± 0.02d	0.65 ± 0.03d	0.34 ± 0.04a	0.76 ± 0.02e
WOE	0.71 ± 0.02c	0.68 ± 0.02b	0.29 ± 0.03c	0.78 ± 0.02c
CE	0.74 ± 0.02b	0.70 ± 0.02a	0.32 ± 0.04b	0.85 ± 0.05b
NCCE	0.76 ± 0.02a	0.71 ± 0.02a	0.27 ± 0.04d	0.87 ± 0.05a
LLE	0.70 ± 0.02d	0.67 ± 0.02c	0.29 ± 0.03c	0.78 ± 0.02d
Stage				
1^st^ flowering	0.75 ± 0.02a	0.71 ± 0.03a	0.31 ± 0.05a	0.84 ± 0.05a
1^st^ fruiting	0.73 ± 0.02b	0.69 ± 0.03b	0.31 ± 0.04b	0.82 ± 0.05b
2^nd^ flowering	0.71 ± 0.02c	0.67 ± 0.02c	0.30 ± 0.04c	0.80 ± 0.05c
2^nd^ fruiting	0.69 ± 0.02d	0.65 ± 0.02d	0.29 ± 0.04d	0.77 ± 0.05d
F-test				
Year (Y)	***	***	***	***
Leafy vegetable (LV)	***	***	***	***
Stage (S)	***	***	***	***
Y × LV × S	ns	ns	ns	ns

Data are presented as means with standard deviation (n= 9). Different letters show significant difference at p<0.05 level. Fv/Fm, photochemical efficiency; ФPSII, photosystem II; NPQ, non-photochemical quenching coefficient; qP, photochemical quenching. FE, fellow eggplant; WOE, welsh onion-eggplant: CE, celery-eggplant; NCCE, non-heading Chinese cabbage-egglant. *** p<0.001; ns, non-significant.

### 3.3 Effect of different leafy vegetable cropping systems on chlorophyll fluorescence parameters

Crop rotation with leafy vegetable cropping systems significantly (*P <* 0.05) affected Fv/Fm, ФPSII, NPQ, and qp (**Table 3**). Among different leafy vegetable rotations, the maximum increase in Fv/Fm, ФPSII, and qP was observed for NCCE cropping system as compared to FE, and the increase was 8.57, 9.23, 25.92, and 14.47%, respectively. At different growth stages, maximum Fv/Fm, ФPSII, and qP were observed at 1st flowering stage, and the increase was 8.69%, 9.23%, and 9.09%, respectively. However, NPQ was higher in FE than other leafy vegetables and increased by 6.89% compared to other leafy vegetables (**Table 4**). The year factor was also significant for chlorophyll fluorescence parameters, and maximum Fv/Fm, ФPSII, and NPQ were observed in 2017, and qP was observed maximum in 2018 (**Table 3**).

**Table 4:** Effect of different leafy vegetable rotation systems on the antioxidant system and soluble protein of eggplant at different growth stages during the year 2017 and 2018.

Treatment	SOD activity (U g^-1^ FW h^-1^)	POD activity (U mg^-1^ protein min^-1^)	PAL (A290 g^-1^ h^-1^)	PPO (0.001ΔA min^-1^)	APX activity Mm/(g. min)	CAT activity (U mg^-1^ protein min^-1^)	Soluble protein (mg g^-1^)
Year							
2017	615.8 ± 175.9b	18.76 ± 4.51b	2758 ± 819.9b	368.5 ± 222.5a	7.96 ± 1.52b	25.89 ± 5.24b	15.82 ± 2.88b
2018	659.3 ± 157.5a	19.29 ± 4.71a	2820 ± 799.6a	324.0 ± 187.0b	8.66 ± 2.02a	27.68 ± 4.16a	16.30 ± 2.81a
Leafy vegetables							
FE	537.0 ± 151.3e	15.20 ± 2.70d	2462 ± 723.6e	307.8 ± 175.9d	7.51 ± 1.80e	23.57 ± 4.05d	14.14 ± 2.14c
WOE	560.5 ± 154.9d	16.39 ± 2.91c	2642 ± 761.4d	332.1 ± 192.0c	7.87 ± 1.90d	25.11 ± 4.03cd	15.26 ± 1.77b
CE	692.3 ± 142.6b	20.38 ± 3.77b	2957 ± 768.2b	369.3 ± 220.9a	8.73 ± 1.62b	28.17 ± 4.06ab	17.68 ± 3.33a
NCCE	743.0 ± 153.0a	23.36 ± 4.51a	3146 ± 819.3a	373.8 ± 227.6a	8.93 ± 1.72a	30.17 ± 4.72a	17.87 ± 2.51a
LLE	654.7 ± 143.7c	19.80 ± 3.70b	2737 ± 795.3c	348.0 ± 205.7b	8.50 ± 1.67c	26.90 ± 4.23bc	15.36 ± 2.19b
Stage							
1^st^ flowering	438.0 ± 57.51d	15.03 ± 2.14d	1635 ± 191.9d	130.4 ± 6.83d	6.28 ± 0.80d	21.81 ± 3.81c	13.00 ± 1.56d
1^st^ fruiting	594.0 ± 125.5c	17.09 ± 3.16c	2686 ± 273.1c	161.3 ± 18.44c	7.63 ± 0.63c	25.65 ± 3.36b	15.52 ± 1.92c
2^nd^ flowering	690.5 ± 107.6b	20.30 ± 3.37b	3082 ± 318.4b	514.0 ± 50.55b	8.46 ± 0.48b	29.51 ± 3.19a	17.43 ± 2.03b
2^nd^ fruiting	827.6 ± 45.81a	23.69 ± 4.06a	3753 ± 240.3a	579.2 ± 69.96a	10.87 ± 0.96a	30.17 ± 3.44a	18.30 ± 2.39a
F-test							
Year (Y)	***	*	***	***	***	***	*
Leafy vegetable (LV)	***	***	***	***	***	***	***
Stage (S)	***	***	***	***	***	***	***
Y × LV × S	**	ns	***	***	***	ns	ns

Data are presented as means with standard deviation (n= 9). Different letters show significant difference at p<0.05 level. SOD, super oxidase; POD, peroxidase; PAL, phenylalanine ammonia–lyase; PPO, polyphenol oxidase; APX, ascorbate peroxidase: CAT, catalase. FE, fellow eggplant; WOE, welsh onion-eggplant: CE, celery-eggplant; NCCE, non-heading Chinese cabbage-egglant. *p < 0.05; **p < 0.01; ***p < 0.001; ns, non-significant.

### 3.4 Effect of different leafy vegetable cropping systems on antioxidant system and soluble protein

Results for the antioxidant enzymes observed are depicted in **Table 4**. The results revealed that WOE, CE, NCCE and, LLE cropping systems used as a crop rotation enhanced the antioxidant enzyme of eggplant. Among different winter leafy vegetable species, NCCE rotation had shown maximum activity of SOD, POD, PAL, PPO, APX, and CAT as compared to FE, and the increase was 38.36%, 53.68%, 27.78%, 21.44%, 18.90%, and 28.00%, respectively. Furthermore, with respect to different growth stages, various enzymatic activities were observed maximum at 2^nd^ fruiting stage than other growth stages, and a significant increase of SOD (47.01%), POD (36.55%), PAL (56.43%), PPO (77.48%), APX (42.22%) and CAT (27.70%) was observed (**Table 4**). Year factor was also significant, and maximum SOD, POD, PAL, APX, and CAT were observed in 2017, whereas maximum PPO was observed in 2018. The interaction effect of three factors (Y × T × S) on SOD, PAL, PPO, APX, and CAT was also significant, whereas POD activity was non-significant.

Leafy vegetable cropping systems significantly influenced the soluble protein content of eggplant leaves (**Table 4**). A maximum increase in soluble protein content was observed for NCCE (26.37%) and CE (25.41%) as compared to FE. A higher amount of soluble protein was observed at different growing stages at 2^nd^ fruiting stage than in other growth stages. The year factor was also significant, and higher soluble protein content was observed in 2018 than in 2017 (**Table 4**).

### 3.5 Effect of different leafy vegetable cropping systems on MDA and H_2_O_2_

Using different Leafy vegetable cropping systems as a crop rotation significantly (*P <* 0.05) reduced MDA and H_2_O_2_ concentration compared to FE (**Table 5**). A higher reduction in MDA concentration was recorded for CE and NCCE as compared to FE, and an increase was 33.09% and 32.84%, respectively. The year factor was also significant, and a higher concentration was observed in 2018 than in 2017. Similarly, H_2_O_2_ was also lower in all leafy vegetable treatments as compared to FE. Compared to FE, the maximum reduction was observed under NCCE (28.52%) and CE (26.59%). Regarding different growth stages, a higher reduction in MDA and H_2_O_2_ concentration was recorded at the 2^nd^ fruiting stage, and a higher reduction of 60.45% and 51.16% in MDA and H_2_O_2_ respectively was recorded at the 2nd fruiting stage compared to the 1st flowering stage (**Table 5**).

**Table 5:** Effect of different leafy vegetable rotation systems on the oxidative markers of eggplant at different growth stages during the year 2017 and 2018.

Treatment	MDA content (nmol g^-1^ FW)	H_2_O_2_ (µmol g^-1^ FW)
Year		
2017	28.00 ± 12.09 b	21.68 ± 6.47a
2018	29.59 ± 11.89a	21.36 ± 6.12a
Leafy vegetables		
FE	36.35 ± 12.53a	25.87 ± 7.23a
WOE	29.30 ± 10.91b	22.81 ± 6.39b
CE	24.32 ± 10.29c	18.99 ± 4.33d
NCCE	24.41 ± 10.59c	18.49 ± 4.35d
LLE	29.61 ± 11.45b	21.43 ± 5.60c
Stage		
1^st^ flowering	17.86 ± 4.01d	13.37 ± 1.60d
1^st^ fruiting	21.58 ± 5.39c	20.73 ± 2.96c
2^nd^ flowering	30.46 ± 5.90b	24.60 ± 4.19b
2^nd^ fruiting	45.30 ± 7.17a	27.38 ± 4.34a
F-test		
Year (Y)	*	ns
Leafy vegetable (LV)	***	***
Stage (S)	***	***
Y × LV × S	ns	ns

Data are presented as means with standard deviation (n= 9). Different letters show significant difference at p<0.05 level. MDA: malondialdehyde; H_2_O_2_: hydrogen peroxide. FE, fellow eggplant; WOE, welsh onion-eggplant: CE, celery-eggplant; NCCE, non-heading Chinese cabbage-egglant. * p<0.05; *** p<0.001; ns, non-significant.

### 3.6 Effect of different leafy vegetable cropping systems on soil chemical properties

Different leafy vegetable cropping systems used as a crop rotation showed a significant (*P <* 0.05) impact on soil chemical properties such as pH, EC, SOM, and soil available nutrients in both years (**Table 6**). A maximum increment in soil pH was recorded in NCCE by (8.01%) and CE (6.61%) compared with FE. The highest increase in pH was observed at 2^nd^ flowering stage as compared to other stages (**Table 6**). The year factor was also significant, and a higher concentration was observed in 2018 than in 2017. The interaction effect of three factors (Y × T × S) on pH was also significant (**Table 6**). Soil EC showed a downward trend after the inclusion of leafy vegetable cropping systems (**Table 6**). WOE and NCCE exhibited the maximum reduction in EC. The highest increase in EC was observed at 1^st^ flowering stage as compared to other stages (**Table 6**). The year factor was also significant, and a higher concentration was observed in 2018 than in 2017. Similarly, leafy vegetables significantly impacted AN, AP, and SOM. The NCCE exhibited a maximum increment in AN (24.31%), AP (14.81%), and SOM (26.37%) compared with the FE. The year factor was significant, with the highest increment in AP and SOM in 2018 than in 2017 (**Table 6**). The highest increase was observed at 2^nd^ fruiting stage as compared to other stages (**Table 6**). However, AK was higher in CE, which increased AK by 9.78% compared to FE. The year factor was significant, with the highest increment in AP in 2017 than in 2018 (**Table 6**). The highest increase was observed at 2^nd^ fruiting stage as compared to other stages.

**Table 6:** Effect of different leafy vegetable rotation systems on soil chemical properties of eggplant at different growth stages during the year 2017 and 2018.

Treatment	pH	EC (μs . cm^-1^)	AN (mg kg^-1^)	AP (mg kg^-1^)	AK (mg kg^-1^)	SOM (g kg^-1^)
Year						
2017	7.49±0.17b	162.14±60.43a	125.97 ± 3.39a	34.37 ± 0.57b	360.58 ± 9.43a	21.08 ± 0.53b
2018	7.51±0.22a	160.36±71.71b	123.19 ± 3.70b	37.67 ± 0.65a	351.32 ± 9.97b	22.93 ± 0.35a
Leafy vegetables						
FE	7.11±0.09d	288.58±11.42a	113.79 ± 2.99c	34.42 ± 1.20b	338.53 ± 3.63d	19.30 ± 0.36e
WOE	7.56±0.06bc	113.80±5.81e	116.13 ± 3.09c	35.19 ± 1.16b	355.06 ± 2.32c	22.06 ±0.0.49c
CE	7.58±0.05b	126.33±7.47c	136.06 ± 3.59b	35.70 ± 1.25b	371.64 ± 2.90a	23.08 ± 0.28b
NCCE	7.68±0.04a	120.59±5.98d	141.46 ± 3.76a	39.52 ± 1.42a	363.89 ±2.23b	24.39 ± 0.33a
LLE	7.57±0.06bc	154.42±4.98b	115.45 ± 3.11c	35.29 ± 1.50b	350.61 ± 1.58c	21.20 ± 0.17d
Stage						
1st flowering	7.49±0.18b	162.32±20.32a	123.77 ± 1.98b	33.75 ± 2.92bc	343.94 ± 2.57d	21.65 ± 0.56c
1st fruiting	7.50±0.20ab	161.58±21.76a	129.79 ± 1.58a	35.49 ± 2.62c	354.35 ± 2.70c	21.54 ± 0.20c
2nd flowering	7.51±0.23a	159.59±22.40b	119.69 ± 1.24c	36.46 ± 2.92b	359.71 ± 2.80b	22.16 ± 0.25b
2nd fruiting	7.50±0.24ab	159.48±22.57b	125.05 ± 1.23b	38.40 ± 2.69a	365.80± 2.61b	22.67 ± 0.44ba
F-test						
Year (Y)	***	***	*	*	*	***
Leafy vegetable (LV)	***	***	***	***	***	***
Stage (S)	*	***	***	***	***	***
Y × LV × S	***	ns	ns	ns	ns	ns

Data are presented as means with standard deviation (n= 9). Different letters show significant difference at p<0.05 level. AN, available nitrogen; AP, available phosphorus; AK, available potassium; SOM, soil organic matter; FE, fellow eggplant; WOE, welsh onion-eggplant: CE, celery-eggplant; NCCE, non-heading Chinese cabbage-egglant. * p<0.05; *** p<0.001; ns, non-significant.

### 3.7 Effect of different leafy vegetable cropping systems on eggplant fresh and dry biomass

Leafy vegetable cropping systems significantly (P < 0.05) increased eggplant’s fresh and dry weight over FE. Both years, the highest increment in eggplant biomass was observed in NCCE and, CE rotation (**Table 7**). However, the highest enhancement was observed in 2018, and the observed increase was 73.05% in NCCE and 62.06% in CE rotation. The year factor also had a significant effect; the highest increase in fresh weight was observed in 2018 compared to 2017 (**Table 7**). Similarly, dry weight was highest in NCCE and CE in both years, which increased by 51.01% in CE and 71.45% in NCCE over FE in 2018.

**Table 7:** Effect of different leafy vegetable rotation systems on fresh and dry weight of eggplant during the year 2017 and 2018.

Treatment	Fresh Weight (g)			Dry Weight (g)		
	2017	2018	Means	2017	2018	Means
FE	351.41±8.18e	342.13±11.48e	346.77±6.89e	140.70±2.95fg	127.66±4.47g	134.18±10.58e
WOE	448.74±9.00d	474.12±8.05cd	461.43±10.94c	163.53±12.03de	174.12±5.69cd	168.83±4.14c
CE	521.35±12.39bc	454.47±15.86ab	537.91±5.49b	187.88±4.06c	192.79±4.27bc	190.34±7.12b
NCCE	553.35±10.49ab	592.08±11.22a	572.72±11.46a	208.40±5.14ab	218.88±5.34a	213.64±7.90a
LLE	370.74±9.03ef	389.28±9.49e	380.01±7.05d	148.23±4.00ef	153.28±3.89ef	150.76±5.30d
Year means	449.12±21.61b	470.42±25.65a		169.75±6.89a	173.35±8.58a	
Tukey HSD test	Treatment	Year	Interaction	Treatment	Year	Interaction
	***	***	NS	***	NS	*

Data are presented as means with standard deviation (n= 3). Different letters show significant difference at p<0.05 level. FE, fellow eggplant; WOE, welsh onion-eggplant: CE, celery-eggplant; NCCE, non-heading Chinese cabbage-egglant. * p<0.05; *** p<0.001; ns, non-significant.

### 3.8 Correlation between plant physiological, biochemical indexes, and soil chemical properties

**Figure 1:**
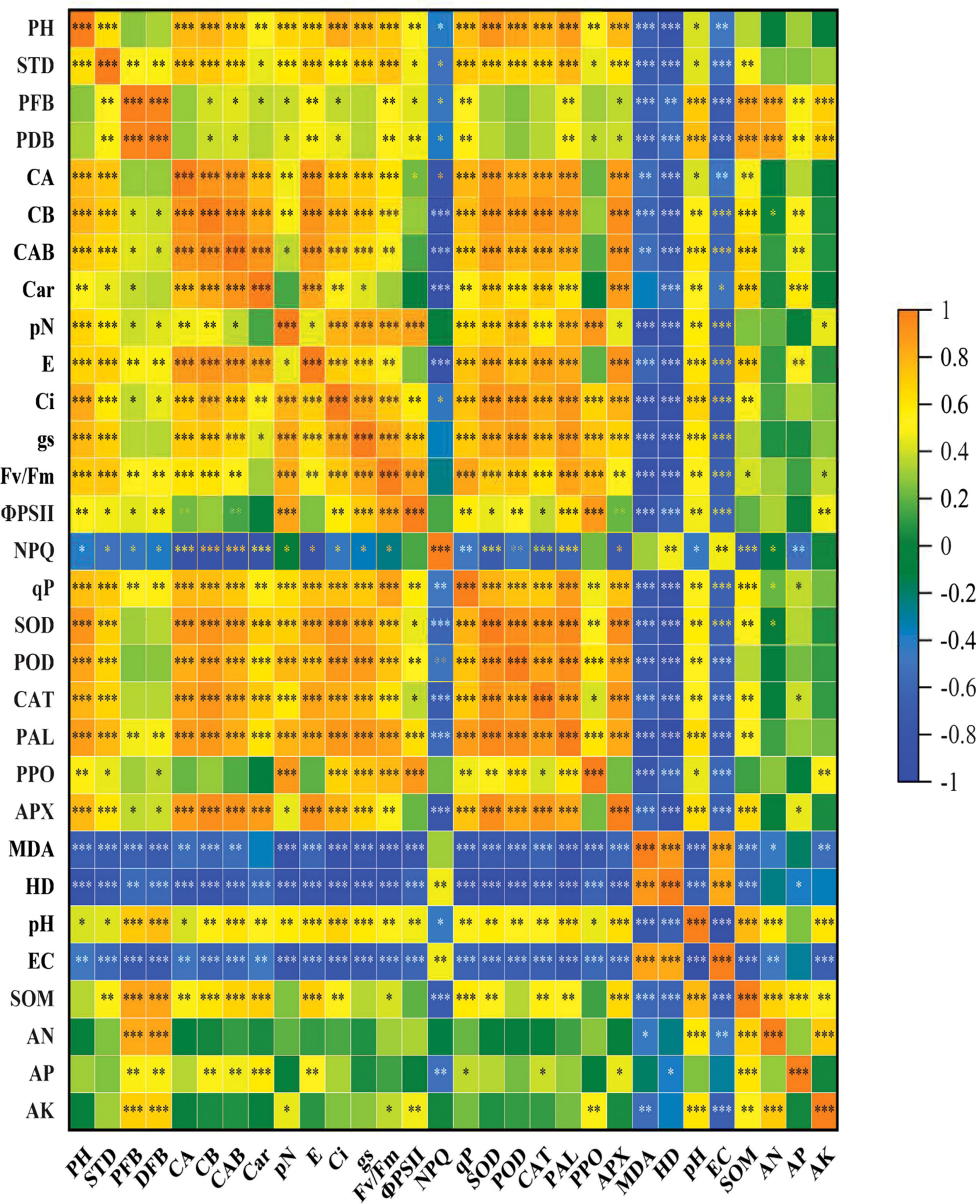
Pearson correlation between eggplant growth, physiological and soil chemical parameters. The brown color represents a positive correlation while the blue color represents a negative correlation. The lighter colors indicate the lower values of the correlation coefficient, while darker colors indicate high positive correlation. PH, Plant height; STD, stem diameter; PFB, plant fresh weight; PDB, plant dry biomass; CA, chlorophyll a; CB, chlorophyll b; CAB, chlorophyll ab; Car, carotenoids; pN, net photosynthesis; E, transpiration rate; Ci, internal CO_2_ rate; gs, stomatal conductance; Fv/Fm, photochemical efficiency; ФPSII, photosystem II; NPQ, non-photochemical quenching coefficient; qP, photochemical quenching; SOD, super oxidase; POD, peroxidase; PAL, phenylalanine ammonia–lyase; PPO, polyphenol oxidase; APX, ascorbate peroxidase; CAT, catalase; MDA, malonaldehyde; HD, hydrogen peroxide; EC, electrical conductivity; AP, available phosphorus; AK, available potassium; SOM, soil organic matter The stars indicate a significant correlation at (*) 0.05, (**) 0.01, (***), and 0.001 levels of significance.

The correlation between various physiological, biochemical, and soil chemical characteristics was examined using the Pearson correlation (**Figure 1**). Based on Pearson correlation PFB, PDB showed a highly significant positive correlation with SOM, AN, AP, and AK. However, these traits showed a highly negative correlation with NPQ, MDA, HD, and EC. Similarly, plant enzymes such as SOD, POD, CAT, PAL, PPO, and APX showed a highly negative correlation with NPQ, MDA, HD, and EC. SOD, POD, CAT, PAL, PPO, and APX positively correlated with SOM. CAT exhibited a positive correlation with AK, while PPO showed a positive correlation with AK.

## 4 Discussion

Our results demonstrated that two years of vegetable cover crops used as a crop rotation significantly impacted soil pH (**Table 6**). It is likely due to the incorporation of dead roots in the soil after harvesting leafy vegetables. Aschi et al. (2017) reported that the residues’ chemical properties could change the soil pH, primarily due to their alkalinity and nitrogen content. Soil organic carbon (SOC) is considered a good soil quality index Ghani et al. (2022d) because it affects soil fertility. Many studies have reported that in crop rotation, below-ground rhizodeposits and root addition are the key factors of carbon accumulation in the soil, accounting for up to 75% of soil organic matter (Jones et al., 2009; Ghani et al., 2022d). Different types of crop stalks, twigs, dead roots as well as fallen leaves are important sources of nutrients Aschi et al. (2017), while leafy vegetables used as crop rotation can scavenge nutrients from the soil, store it in their residues and return it to the soil for the next crop through root decomposition and improved SOM (Dorissant et al., 2022; Ghani et al., 2022b). Kong and Six (2012) reported that living cover crops significantly improved soil organic matter compared to bare fallow due to the rhizodeposition of low molecular carbon into the soil. The rhizodeposition rate decreases with plant age, but the addition of mature roots into the soil as residue acts as a microbial substrate, thus increasing MBC as well as soil organic matter (Chahal and Van Eerd, 2020). In line with this concept, NCCE and CE modify the soil environment (**Table 6**) by improving soil nutrients and SOM through root exudates and dead roots, which were available for eggplant. Our results align with the findings of Ali et al. (2021), where different winter leafy vegetables cover crops used as a crop rotation can enhance soil nutrient availability and SOM by incorporating plant and root residue.

It was observed that eggplant grown on soils after the leafy vegetable crop rotation exhibited increased growth in terms of plant height, stem diameter, and greater biomass (**Tables 1**, **7**) and reflected in significantly increased yield (Ghani et al., 2022b) compared with mono-cropping for two years. Higher eggplant growth and biomass production directly correlated to improved soil chemical properties, evident from the positive correlation between plant growth and soil chemical parameters (**Figure 1**). Similar findings were reported by D’Acunto et al. (2018), where pea-maize crop rotation improved maize biomass which was interlinked with different soil properties.

Furthermore, increased growth and yield in eggplant-leafy vegetable cropping systems were correlated with enhanced photosynthesis and gas exchange, which was confirmed by the positive correlation between eggplant growth and photosynthetic parameters (**Figure 1**). Earlier, it has been reported that mono-cropping or intercropping systems negatively influence the photosynthesis and growth of plants (Yao et al., 2017). Mono-cropping results in the depletion of soil microbial population and mineral status. Mono-cropping of cucumber reduces growth and yield by declining beneficial microbial populations in the soil, and crop rotation significantly increases the yield (Wu and Wang, 2007). Reduced photosynthesis directly results from the restricted stomatal conductance and internal CO_2_ concentrations in mono-cropped eggplant. It is believed that increased stomatal conductance leads to the maintenance of CO_2,_ reflected in increased photosynthetic rate (Ahanger et al., 2018). Reports discussing the role of leafy vegetables-eggplant cropping systems in the protection of the photosynthetic system, growth, and yield are not available. The present investigation shows the protection of available mineral status and soil health due to crop rotation with leafy vegetables. Reduced photosynthesis in the mono-cropping or intercropping system results in declined production of energy, carbohydrate metabolism and chlorophyll production (**Table 2**), (Su et al., 2014). Crop rotation improves soil physical and chemical environment (**Table 6**), including water holding capacity and aeration, and ultimately increases plant growth attributes like root growth and nutrient foraging. In the wheat-peanut crop rotation system, increased nitrogen uptake and allocation resulted in greater chlorophyll synthesis and photosynthesis rate (Liu et al., 2019). Moreover, increased pigment synthesis and photosynthesis in the crop rotation system were linked with improved PSII functioning. Relative to FE, Fv/Fm, ФPSII, and qP increased in all cropping systems while NPQ exhibited a reduction (**Table 3**). Photosystem functioning was considerably enhanced during both experimental seasons due to crop rotation with WOE, CE, NCCE, and LE.net Increased fluorescence parameters reflect photosynthetic regulation through non-stomatal modulations (Ahanger et al., 2020). The present study envisaged that both stomatal and non-stomatal enhancements in the different cropping systems influenced the growth and yield performance of eggplant. Increased access to external CO_2_ and reduced accumulation of toxic radicals in intercropping systems significantly contributed to the functioning of photosynthetic machinery (Yao et al., 2017).

Increased growth and photosynthesis in plants raised in the crop rotation system are due to reduced oxidative damage in them (**Table 5**), resulting in a significant enhancement in the structural and functional integrity of membranes and cells (Liu et al., 2019). Reduced generation of reactive oxygen species prevents the oxidative effects on the membranes reflecting in the maintenance of plant functioning (Ahanger and Agarwal, 2017b). In the present study, eggplant grown on soils following crop rotation with different leafy vegetables also exhibited reduced lipid peroxidation due to a significant decline in the accumulation of hydrogen peroxide during both years. Our findings were further supported by a negative correlation among growth, photosynthesis, and oxidative markers (**Figure 1**). The declining oxidative effects of mono-cropping due to the growth of leafy vegetables were observed due to up-regulation of APX and CAT activities in them during both years and at both developmental stages (**Table 4**). Relative to FE, activities of APX and CAT increased in seedlings grown after leafy vegetable crops, with the maximal increase in plants grown in NCCE system at both developmental stages. APX and CAT act on the same substrate, i.e., hydrogen peroxide but at different sites, with APX eliminating excess hydrogen peroxide from chloroplast while CAT from the cytosol (Ahmad, 2010). Increased APX activity due to crop rotation with leafy vegetables strengthened the key radical scavenging pathways, including ascorbate-glutathione in chloroplasts leading to increased protection of major cellular pathways, including photosynthesis (Ahanger and Agarwal, 2017a; Ghani et al., 2022a). Up-regulation of the activities of APX due to crop rotation prevents the formation of toxic hydroxyl radicals by assisting in the maintenance of redox homeostasis and the electron donors, including ascorbate and glutathione (Khan and Khan, 2014). Greater ascorbate and glutathione content due to improved ascorbate-glutathione functioning maintains the electron transport in chloroplasts and mitochondria (Nahar et al., 2016).

In addition to this protein content of eggplant increased significantly due to crop rotation with leafy vegetables. During both years, maximal protein content was reported at the second fruiting and second flowering stages. During both years of experimentation, the influence of leafy vegetable crop rotation on eggplant protein content slowly increased from 2017 to 2018 and showed an increasing trend with the developmental stage (**Table 6**), indicating the development of beneficial proteins under a crop rotation system. Proteins form an important nutritional component of plants, particularly in vegetables, and help plants maintain growth and development under different growth conditions (Lee and Yaffe, 2016). Proteins assist in signaling and maintaining development from seed germination to flowering. Plants have specific proteins maintaining key cellular functioning like photosynthesis, signaling, and response elicitation. Intercropping and crop rotation systems have been proposed to influence plant development by modulating physiology and biochemistry and reducing disease incidence; however, the effect has been reported to be species-dependent (Crow et al., 2000; Ghani et al., 2019a). Such beneficial effects of crop rotation with leafy vegetables can be due to the significant decline in the accumulation of allelopathic compounds within the eggplant rhizosphere, thereby declining the growth through auto-allelopathy (Cheng and Cheng, 2015). However, eggplant crop rotation with leafy vegetables may have reduced the accumulation of allelochemicals and improved the synthesis of some specific proteins. Proteins mediate specific defense pathways in plants (Chuang et al., 2016). In addition, the activity of PAL is stimulated significantly due to crop rotation with vegetable crops.

PAL regulates the synthesis of secondary metabolites in plants. Increased PAL activity has been reported to contribute to greater stress tolerance by enhancing antioxidant potential (Ahanger and Agarwal, 2017b). Increased PAL activity due to crop rotation with leafy vegetables may improve eggplant metabolite content with significant health benefits (Gürbüz et al., 2018). Eggplant is rich in some key metabolites contributing to its functional and pharmaceutical properties (Rodriguez-Jimenez et al., 2018). Increased PAL and PPO activity (**Table 4**) under crop rotation with different vegetables justify the beneficial effect on secondary metabolism. The accumulation of secondary metabolites is regulated by PPO, which does this by oxidizing phenols. This, in turn, mediates fruit harvesting and resistance to pathogens. On the other hand, it has been reported that silencing PPO makes pathogen infection more likely by modifying the accumulation of phenolic compounds and their derivatives (Araji et al., 2014). However, through metabolomics increase in individual metabolites can be assessed to unravel the exact mechanisms involved.

## 5 Conclusion

Conclusively results of the study, which was carried out underneath a plastic shed using sustainable practices, indicated that different leafy vegetable species could be successfully used to minimize external inputs without a reduction in yield. The study was carried out within the context of the transition of agricultural practices toward the cultivation of sustainable vegetables. Conclusively, crop rotation of eggplant with leafy vegetable cropping systems, including WOE, CE, NCCE, and LE, exhibited greater yield and growth through improving soil chemical properties, modulation in the photosynthetic efficiency and gas exchange parameters. Increased activity of antioxidant enzymes imparted reduced oxidative damage by lowering the generation of reactive oxygen species. In addition, crop rotation with leafy vegetables may have regulated the metabolism of secondary metabolites through the upregulation of PAL and PPO. By modulating ROS and altering the activity of antioxidant enzymes, NCCE, and CE were more effective in improving growth and yield than other leafy vegetable species assessed in this study, including fallow eggplant. This was determined by comparing their results to those of other leafy vegetable plants. Further studies at transcriptomic, metabolomic, and molecular levels would be helpful in unraveling the exact mechanisms of the above findings.

## Data availability statement

The raw data supporting the conclusions of this article will be made available by the authors, without undue reservation.

## Author contributions

ZC: Scientific concept, foundation, conceptualization, research idea, experimental guiding, revising, editing, and corresponding author, XC: review, foundation, editing, proof reading of the entire manuscript and co-corresponding author, MIG: Performing the field experiment, investigation, experimental guiding, data collection, methodology, and writing the draft manuscript, MAt, AL, MAl and MN: Investigation, accurateness of data analysis, and correction. MIG and MAh analyzed the data, interpreted the results, and wrote the paper with input from all authors. All authors contributed to the article and approved the submitted version.
